# Prevalence of Zinc Deficiency in 3-18 Years Old Children in Shiraz-Iran

**Published:** 2011-01-01

**Authors:** S M Dehghani, P Katibeh, M Haghighat, H Moravej, S Asadi

**Affiliations:** 1 Gastroenterohepatology Research Center, Department of Pediatric Gastroenterology, Nemazee Hospital, Shiraz University of Medical Sciences, Shiraz, Iran; 2 Department of Pediatrics, Shiraz University of Medical Sciences, Shiraz, Iran

**Keywords:** Zinc, Children, Malnutrition, Deficiency, Iran

## Abstract

**Background:**

Zinc is an essential trace element with a prominent role in human nutrition. Zinc deficiency has been linked to growth retardation, hypogonadism in males, and lack of sexual development in females. It ranges from 50% in sub-Saharan Africa to 5% in high income countries. The aim of this study is to evaluate the prevalence of zinc deficiency in healthy children in Shiraz, southern Iran.

**Methods:**

In this study, 902 children aged 3-18 years old were randomly sampled for serum zinc level. Age, sex, weight, height, BMI, stunting and wasting indices were also recorded. With atomic absorption spectrophotometry method, the serum level of zinc less than 70 µg/dL was considered as deficient.

**Results:**

Mean serum level of zinc was 122.3±55 µg/dL. The prevalence of zinc deficiency was 7.9%. There was no relationship among serum zinc level and age, sex, height, weight or BMI, but mild wasting (weight for age) and mild stunting (height for age) were significantly more prevalent among zinc deficient children compared to children with normal or high level of zinc.

**Conclusion:**

Zinc deficiency in Shiraz is not as prevalent as other areas of Iran. It was significantly more frequent among stunted and wasted (malnourished) children. Difference in soil zinc level, recent wide prescription of zinc supplements by pediatricians and especial pattern of nutrition, considered as possible factors responsible for lower prevalence of zinc deficiency in Shiraz, deserve more investigations.

## Introduction

Zinc is an essential trace element that has a prominent role in human nutrition and health. It is required for the catalytic activity of approximately 100 enzymes and having a significant role in cell division, protein synthesis, wound healing, and immune function.[1] Daily intake of zinc is required especially during childhood, adolescence and pregnancy because the body has no specialized zinc storage system.[2]

A conservative estimate suggests that 25% of the world population is at risk of zinc deficiency.[3] According to Disease Control Priorities in developing countries second edition (2006), zinc deficiency ranges from 79% in the south of Asia to 5% in high income countries. In the Middle East and North Africa, its prevalence is 46%, in Eastern Asia and Pacific 7%, and in Latin America and Caribbean 33%.

Zinc deficiency has been linked to growth retardation, weight loss, intercurrent infections, hypogonadism in males, lack of sexual development in females, rough skin, poor appetite, delayed wound healing, acne, poor immune system, diarrhea, pneumonia and taste abnormalities.[3][4] Growth, pneumonia, diarrhea, malaria and neuropsychiatric performance respond significantly to improved zinc status.[5][6][7]

Measurement of zinc concentration in the plasma has been shown to be useful in identifying children who are likely to grow better in response to zinc supplementation.[6] Hair zinc concentration, although it may propose useful data, is less defined.[8] This is also the same for what theoretically may be considered a more putative biomarker, i.e. the activity of a zinc-dependent enzyme.[9]

Assessment of the prevalence of zinc deficiency in different areas is a useful guide to treat the children at risk of growth retardation with sufficient supplementation of zinc.

The aim of this study is to evaluate the prevalence of zinc deficiency in healthy children in Shiraz, south of Iran.

## Materials and Methods

In a cross-sectional study, carried out during 9 months between March and December 2008, 902 children aged 3-18 years were sampled for measurement of their serum zinc level. They were selected in a systematic random sampling method by referring to their home in different areas of Shiraz based on educational division. Exclusion criteria were vegetarian diet, diarrhea, history of chronic liver and renal diseases, diabetes, malignancy, sickle cell anemia, acrodermatitis enteropathica and recent zinc supplementation in last 6 months. After explaining the goal of the study for the parents or legal guardians and giving written informed consent, permission was given to take 4-5 mL of blood from their children. Age, sex, weight, and height were all recorded. As serum zinc is thought to be higher in the morning, all the samples were taken in the morning.[10]

Serum zinc level was measured with atomic absorption spectrometry in Shiraz Gastroenterohepatology Research Center Laboratory in Nemazee Hospital affiliated to Shiraz University of Medical sciences. It has been suggested that plasma level of 70-158 µg/dL (11-19 µmol/L) is normal, and less than this value is considered as zinc deficient.[11] Although various cut off points for zinc deficiency are considered in different studies, in this study, as mentioned, serum level of less than 70 was considered as zinc deficiency. Body mass index (BMI) was calculated and plotted on BMI diagram for age percentiles (boys and girls) from Nutritional Center for Health Statistics (2000). At the same time, severity of malnutrition [wasting (weight for age), and stunting (height for age)] was measured by Waterlow and Gomez indices.[12][13]

The children were divided into three groups of 3-6, 6-12 and 12-18 years of age. As to BMI index, they were categorized into four groups: BMI<5th percentile as underweight, between 5th and 84th percentile as normal weight, between 85th and 95th as at risk for overweight and >95th percentile as overweight. In order to evaluate the severity of malnutrition, the children were divided into normal, mild, moderate and severe groups for wasting and stunting indices (Table 1).

**Table 1:** Severity of malnutrition in children according to weight for age and height for age

**Grade of ** **malnutrition**	**Weight for ** **age (wasting)**	**Height for ** **age (stunting)**
0, normal	>90	>95
1, mild	75-90	90-95
2, moderate	60-74	85-89
3, severe	<60	<85

The data were analyzed with SPSS software (version 15, Chicago, IL, USA). All the factors were tested for distribution model. One way ANOVA and t tests were used for data with normal distribution. P-values less than 0.05 were considered significant.

At the end of study, zinc deficient children were guided to take adequate supplementation of zinc and those who were underweight, wasted or stunted were closely followed and treated. The study was performed in accordance with the principles of the Declaration of Helsinki 2000 and was approved by the local Ethics Committee of the Shiraz University of Medical sciences.

## Results

Out of 902 subjects, 496 (55%) were male. Group 1 (3-6 years of age) consisted of 173 children (19%), group 2 (6-12 years of age) of 369 (41%) and group 3 (12-18 years of age) of 360 (40%). The mean serum zinc level in 3-6 years old children was 117.7±45.1 µg/dL, in 6-12 years of age 121.8±64.3 µg/dL, and in 12-18 years of age 124.9±49.8 µg/dL. Mean Zinc level in all age groups was 122.3±55 µg/dL. In boys, this value was 119.6±43.9 µg/dL and in girls 125.7±67.2 µg/dL. As to gender, there was no significant difference in serum zinc level (p=0.142).

The prevalence of zinc deficiency was defined as serum zinc level less than 70 µg/dL that was 10.9% in 3-6 years of age, 8.9% in 6-12 years of age and 5.4% in 12-18 years of age and in all age groups it was 7.9%. There was no significant association between zinc deficiency and age (p=0.142). The data are demonstrated in Table 2.

**Table 2:** Relation of zinc deficiency and different variables

**Variable**	**Prevalence of zinc** **deficiency (%)**	**P-value**
Age (years):		0.441
3-6	10.9	
6-12	8.9	
12-18	5.4	
Total	7.9	
Sex:		0.142
Boys	8.1	
Girls	7.8	
Stunting (height for age):		0.029^a^
Normal	50.7	
Mild	47.8	
Moderate	1.5	
Wasting (weight for age):		0.033^a^
Normal	49.3	
Mild	44.8	
Moderate	6.0	
BMI (adjusted for age):		0.207
Under weight	10.2	
Normal weight	7.8	
At risk for overweight	4.8	
Over weight	5.8	

^a^ significant

As to BMI, 16% of the subjects were underweight, 73% were normal, 5% at risk for overweight and 6% overweight. Serum zinc level was not significantly related to BMI, height or weight either. In the underweight group, mean zinc level was 125.9±90.3 µg/dL, in the normal weight 122.2±46.7 µg/dL, in at risk for overweight 119.4±51.6 µg/dL and in overweight 118.8±37.3 µg/dL. The prevalence of zinc deficiency was 10.2%, 7.8%, 4.8%, and 5.8% in underweight, normal weight, at risk for overweight and overweight, respectively (p=0.207).

However, the prevalence of mild wasting and stunting was significantly higher in zinc deficient children compared to children with normal or high zinc level. In the zinc deficient group, 44.8% were mildly and 6% moderately wasted (p=0.033). As to height for age in zinc deficient children, 47.8% were mildly and 1.5% moderately stunted (p=0.029).

## Discussion

The history of recognition of biological role of zinc refers back to the nineteenth century, by the late 1950s it was accepted that zinc was a necessary micronutrient for humans but the significance of human zinc deficiency due to nutritional origin was not really elucidated.[9] In 1961, Prasad et al. suggested a major role for zinc as an etiologic factor of adolescent nutritional dwarfism in mid-Eastern countries.[14] Double-blind, controlled, randomized studies have assessed growth velocity associated with modest dietary zinc supplements and have confirmed the effect of zinc supplementation in increasing height and weight.[5][6][15][16] In Iran, Hakimi et al., Ebrahimi et al., and Ronaghi et al. in three different studies again proved this theory.[17][18][19] The prevalence and burden of zinc deficiency is quite different all over the world, according to disease control priorities in developing countries (2006), the prevalence of zinc deficiency in the Middle East is about 46%. There are various data from different areas in Iran. In Tehran, it was reported 28%,[20] 30.1% [21] and 85.5%[22] in three different studies. Fesharakinia et al. reported that, zinc deficiency in Khorasan is 28.1%;[23] however, cut off point for serum zinc ranged 70-100 µg/dL in these different studies. Sharifi et al. showed that the prevalence of zinc deficiency was different in 23 provinces of Iran.[21] In present study, serum zinc level less than 70 was considered to be deficient;[11] however, the prevalence of zinc deficiency was 7.9%. Perhaps, the difference of serum zinc level in different areas can be attributed to soil zinc concentration, but Qin et al. in 2009 found that the prevalence of zinc deficiency and stunting was not significantly high in children of rural areas with low soil zinc concentrations.[24] Difference in dietary intake, excessive supplementation with zinc in some areas along with different soil zinc level and cut off point of serum zinc level may be other reasons to explain various statistics for zinc deficiency. In one study, higher intake of phytate relative to zinc, explained for higher frequency of zinc deficiency and stunting in children.[8]

In this study, mean serum zinc level was 122.3±55 µg/dL whereas in different studies in Iran it was about 95.2±177 µg/dL in ones study,[20] 54.6±23.3 µg/dL in another study in Tehran,[22] and 87.7±32 µg/dL in Birjand.[23] In the present study, there was no significant relationship between sex and serum zinc level but zinc deficiency was slightly more prevalent among boys (8.1% compared to 7.8% in girls). This is compatible to what Arvanitidon et al. in Greece and Ohtake et al. in Japan reported.[25][26]

No significant relationship was found in our study between zinc level and height, weight or BMI, but prevalence of mild wasting and mild stunting was significantly higher in zinc-deficient children compared to children with normal or high zinc level. Costa et al. in Brazil reported prevalence of zinc deficiency to be 74.3%, but no relation was found between zinc, height or weight.[27] Other studies confirmed the effect of zinc on height and weight and correlation between them.[25][26] As to age, no relationship was detected with serum zinc level. This is similar to the reports of Ohtake et al.,[26] in Japan and Fesharakinia et al.[23] in Iran, but it is contrary to the results of Alarcon et al. study in Venezuela.[28]

According to WHO/UNICEF/IAEA/IZiNCG, when the prevalence of zinc deficiency is greater than 20%, intervention to improve zinc status is recommended and if the prevalence of inadequate intake is greater than 25%, the risk of zinc deficiency is considered to be elevated.[29] Although vitamin and mineral supplements can help infants and toddlers with special nutrient needs or marginal intake achieve adequate intakes, care must be taken to ensure that supplementation does not lead to excessive intakes.[30]

So, it is recommended to revise the diet of Iranian children in order to provide sufficient amounts of zinc supplement in all areas of Iran, but dietitians should encourage primary source of zinc in food rather than supplementation, especially after 6 months of age when breast feeding does not provide the sufficient amount of zinc any more.

We concluded that zinc deficiency in Shiraz is not as prevalent as other areas of Iran. It was significantly more frequent among stunted and wasted (malnourished) children. Difference in soil zinc level, recent wide prescription of zinc supplements by pediatricians and especial pattern of nutrition, considered as possible factors responsible for lower prevalence of zinc deficiency in Shiraz, deserve more investigations.
